# Contributions of Long-Read Sequencing for the Detection of Antimicrobial Resistance

**DOI:** 10.3390/pathogens13090730

**Published:** 2024-08-28

**Authors:** Roberto Sierra, Mélanie Roch, Milo Moraz, Julien Prados, Nicolas Vuilleumier, Stéphane Emonet, Diego O. Andrey

**Affiliations:** 1 Infectious Diseases Division, Department of Medicine, Geneva University Hospitals and Faculty of Medicine, 1205 Geneva, Switzerland; roberto.sierra@unige.ch (R.S.);; 2 Department of Microbiology and Molecular Medicine, Faculty of Medicine, University of Geneva, 1206 Geneva, Switzerland; 3 Division of Laboratory Medicine, Diagnostics Department, Geneva University Hospitals and Faculty of Medicine, 1205 Geneva, Switzerland; 4 Infectious Diseases Division, Institut Central des Hôpitaux (ICH), Valais Hospital, 1951 Sion, Switzerland; 5 Bioinformatics Support Platform, Faculty of Medicine, University of Geneva, 1206 Geneva, Switzerland

**Keywords:** long-read sequencing, Oxford Nanopore Technologies, carbapenemase, *Klebsiella*

## Abstract

Background. In the context of increasing antimicrobial resistance (AMR), whole-genome sequencing (WGS) of bacteria is considered a highly accurate and comprehensive surveillance method for detecting and tracking the spread of resistant pathogens. Two primary sequencing technologies exist: short-read sequencing (50–300 base pairs) and long-read sequencing (thousands of base pairs). The former, based on Illumina sequencing platforms (ISPs), provides extensive coverage and high accuracy for detecting single nucleotide polymorphisms (SNPs) and small insertions/deletions, but is limited by its read length. The latter, based on platforms such as Oxford Nanopore Technologies (ONT), enables the assembly of genomes, particularly those with repetitive regions and structural variants, although its accuracy has historically been lower. Results. We performed a head-to-head comparison of these techniques to sequence the *K. pneumoniae* VS17 isolate, focusing on *bla*_NDM_ resistance gene alleles in the context of a surveillance program. Discrepancies between the ISP (*bla*_NDM-4_ allele identified) and ONT (*bla*_NDM-1_ and *bla*_NDM-5_ alleles identified) were observed. Conjugation assays and Sanger sequencing, used as the gold standard, confirmed the validity of ONT results. This study demonstrates the importance of long-read or hybrid assemblies for accurate carbapenemase resistance gene identification and highlights the limitations of short reads in the context of gene duplications or multiple alleles. Conclusions. In this proof-of-concept study, we conclude that recent long-read sequencing technology may outperform standard short-read sequencing for the accurate identification of carbapenemase alleles. Such information is crucial given the rising prevalence of strains producing multiple carbapenemases, especially as WGS is increasingly used for epidemiological surveillance and infection control.

## 1. Introduction

Antimicrobial resistance (AMR) is a major public health concern, undermining the effectiveness of antibiotic treatments and posing a threat to global healthcare systems and modern medicine. Bacterial whole-genome sequencing (WGS) as a surveillance strategy for AMR is a highly accurate and comprehensive method as it allows for detecting and monitoring the presence and spread of resistant pathogens by genomic characterization [1]. This approach provides detailed insights into the genetic mechanisms underlying resistance and helps in tracking the evolution and dissemination of AMR genes. It also enables the identification of genetic elements associated with resistance in a bacterial genome, including known and novel resistance genes and alleles, as well as mobile genetic elements such as plasmids and transposons [2,3].

Short- and long-read sequencing are two primary technologies used in genomics, each with its strengths and weaknesses. Short-read sequencing (50–300 base pair length), typically based on Illumina sequencing platforms (ISPs), generates extensive coverage (large number of reads and amount of data) and is recognized for its high accuracy. These features allow excellent detection of single nucleotide polymorphisms (SNPs) and small indels. The main disadvantage is the limited read length, which makes it impossible to fully assemble genomes due to structural variants and low-complexity regions such as tandem repeats and GC-biased regions or regions containing the same mobile genetic element [4,5]. In contrast, long-read sequencing, typically based on platforms like Oxford Nanopore Technologies (ONT) and Pacific Biosciences, generates read lengths of thousands or more base pairs, and has been recognized as a reliable strategy for assembling genomes, especially those with repetitive regions. Better at spanning over complex genomic regions, such as large structural variants, duplications, and translocations, it also allows for complete plasmid assemblies and circularization [6,7]. Nevertheless, although ONT previously had lower accuracy, recent advancements in chemistry have resulted in higher yields and comparable Phred scores to the ISP [8,9,10]. Finally, hybrid assemblies, merging data from both short- and long-read sequencing, combine both techniques to leverage their advantages but not without challenges of their own [11,12].

As part of our surveillance program, we sequenced the *K. pneumoniae* VS17 isolate using short- and long-read techniques and identified discrepancies in resistance gene allele identification, with discordant NDM alleles across assemblies from each technique. We further investigated these carbapenemase allele inconsistencies by comparing short-read-only, long-read-only, and hybrid genome assemblies against Sanger sequencing, considered the gold standard.

## 2. Methods

### 2.1. Bacterial Isolates, Identification, and Carbapenemase Detection

As part of our institution’s surveillance strategy for carbapenemase-producing *K. pneumoniae* and other Enterobacterales species, we processed ano-rectal MDRO vigilance rectal swabs (eSwab™, Copan, Murrieta, CA, USA) by plating on selective CHROMagar and mSuperCARBA™ (CHROMagar, Saint-Denis, France) plates, followed by identification by VITEK-MS (BioMérieux, Marcy-l’Étoile, France) mass spectrometry. Isolates were further tested using the carbapenemase GenXpert (Cepheid, Macquarie Park, Australia) platform. The *K. pneumoniae* VS17 strain was then selected. The azide-resistant *E. coli* J53 strain was available at our lab and used for conjugation assays.

### 2.2. DNA Extractions and Sequencing

The bacterial strain was cultured in LB broth at 37 °C until O.D. ~0.7, and then 1.5 mL of culture was collected and centrifuged. Bacterial pellets were washed with PBS, supernatants were discarded, and then pellets were frozen at −80 °C. DNA was extracted using the DNeasy Blood and Tissue Kit (Qiagen, Hilden, Germany) and quantified using a Qubit fluorometer (ThermoFisher Scientific, Waltham, MA, USA). For ISP short-read sequencing, genomic DNA was quantified with a Qubit fluorometer (Life Technologies, Carlsbad, CA, USA). The Tagmentation kit was used for the library preparation with 200 ng of DNA as input. Library molarity and quality was assessed with Qubit and TapeStation using a DNA high-sensitivity chip (Agilent Technologies, Santa Clara, CA, USA). The library was sequenced on a HiSeq 4000 for 100 paired-end reads. For long-read sequencing, *K. pneumoniae* DNA was extracted using the Promega HMW DNA Extraction kit (Madison, WI, USA). The ONT (Oxford Nanopore Technologies, Oxford, UK) Nanopore Native Barcoding Kit 24 (SQK-NBD112.24) was used, and the library was loaded onto an R10.4 (FLO-MIN112) flow cell and sequenced for 72 h. Transconjugant *E. coli* DNA was extracted as above, and libraries were prepared using the Nanopore Native Barcoding kit 24 V14 (SQK-NBD114.24) and then sequenced on an R10.4.1 (FLO-MIN114) flow cell.

### 2.3. WGS Data Analysis and Typing

Short-read fastq files were assembled using SPAdes (v.3.15.4) and hybrid assemblies were produced with Unicycler (v.0.5.0, Racon v.1.5, makeblastdb/tblastn v.2.4.0+). ONT long-read sequencing fast5 output files were base-called using Guppy (v. 6.1.2+e0556ff93, minimap2 v. 2.22-r1101) with the super accuracy model (sup) on the HPC servers at the University of Geneva. The resulting fastq files were assembled using Flye (v. 2.9.1) assembler software. The Flye assemblies were corrected with the short-read ISP data using three rounds of Pilon (v. 1.24) or long-reads using Medaka (v.1.7.0). Raw read quality control was performed with in-house R scripts to calculate the number of reads, mean, minimum, median, and maximum length, N50, total number of bases, ambiguous bases, and average read quality. Similarly, assemblies were queried for the number of contigs produced, total assembly length, maximum and minimum contig length, number of ambiguous bases, and N50. The software developed by the Center for Genomic Epidemiology was used in local clusters to identify MLST, resistance genes (ResFinder v.4.6.0), and plasmid types (PlasmidFinder v.2.1.0). These databases are among the most comprehensive for typing clinical isolates, e.g., the PlasmidFinder database contains almost 500 different plasmid types for Gram-positive and -negative bacteria and is regularly updated [13].

### 2.4. Conjugations Assays

Mating experiments were carried out in LB broth by mixing stationary-phase cultures in a ratio of 1:3 (donor/recipient) using the VS17 (*K. pneumoniae*) isolate carrying *bla*_NDM-1_ and *bla*_NDM-5_ plasmids as the donor and *E. coli* J53 (azide-resistant derivative of *E. coli* K12) as the recipient strain. After overnight co-incubation at 37 °C, the cells were plated on UTI ChromSelect agar (Millipore, Darmstadt, Germany) supplemented with sodium azide (150 mg/L) and meropenem (1 mg/L). The presence of the plasmids in transconjugants was validated as described below.

### 2.5. Validation of bla_NDM_ Alleles

Primers amplifying specifically the two *bla*_NDM_ regions were designed based on long-read sequencing data: up-NDM-VS17-contig1 (5′-GGGACAAGAAAATCTCTTTTCTGG-3′) and up-NDM-VS17-contig3 (5′-AGGTTCATCACTTTATGGATACCG-3′) were combined with the down-NDM-VS17 (5′-CCATGGCATCGAGATCATCC-3′). PCR products were sequenced by Sanger sequencing (Fasteris, Switzerland) to confirm the allele identity in the *K. pneumoniae* VS17 isolate. Similarly, the *E. coli* transconjugants were initially screened by PCR amplification of each NDM region. One transconjugant was then sequenced using Nanopore long-read technology to corroborate the structure and size of the plasmid. Alignments were performed using Minimap2 as implemented in Geneious Prime v2024.0.5.

## 3. Results

### 3.1. Sequencing Output

Short and long reads were obtained for *K. pneumoniae* VS17 with sequence coverage ranging from 230× to 349× for the ISP and ONT, respectively, considering an estimated genome size of 5.5 M bases. These coverages were obtained with >12 M reads (N50 100 bp) with the ISP and >0.6 M reads (N50 6802 bp) with ONT. Base calling quality was as expected for each technology; ISP quality scores almost reached Q40 (i.e., 99.99% accuracy) and ONT Q30 (i.e., 99.9% accuracy) values (Table 1).

**Figure 1:**
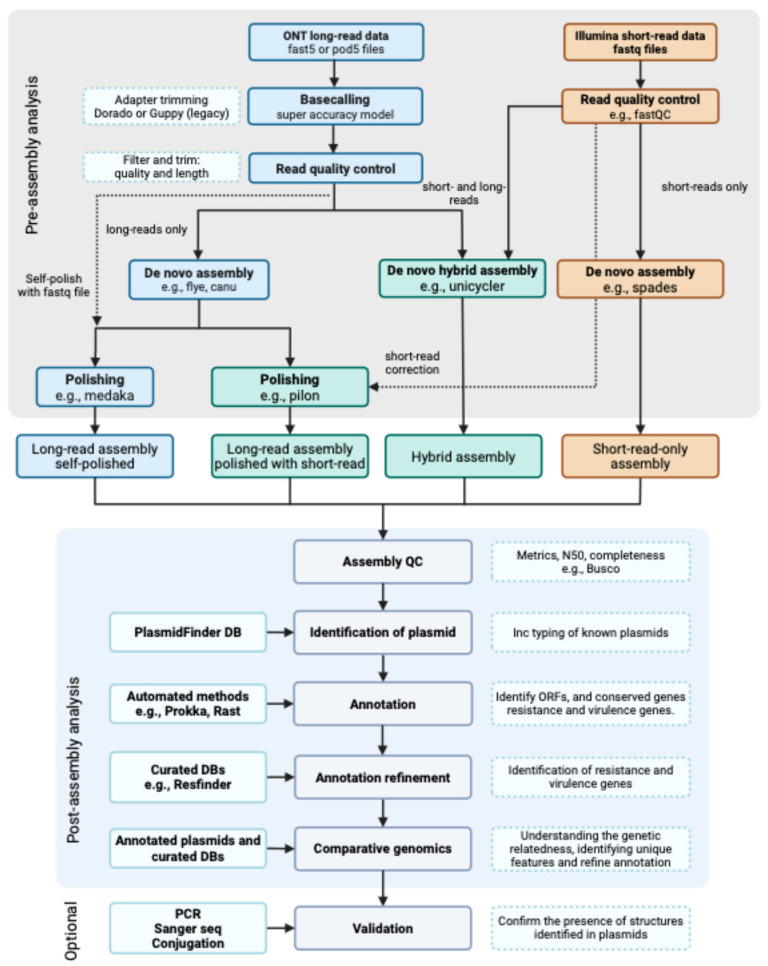
Overview of the analysis pipeline from raw sequencing output to assembly and annotations. The steps in blue (top left) lead to long-read only assembly, in green (top center) combined short- and long-read assemblies, and in orange (top right) short-read-only assemblies.

Assemblies were performed following the analysis pipeline in Figure 1. Four assemblies were obtained following different analyses involving short- and long-read-only assemblies, hybrid assembly, and polishing methods with short- and long-read data. The resulting summary assembly statistics are shown in Table 2. A SPAdes assembly using ISP reads only produced 277 contigs that were consolidated into 250 scaffolds by adding 2610 ambiguous positions. The maximum scaffold length obtained was 658,511 bp. Long-read assemblies produced five circular contigs representing the bacterial chromosome and probable plasmids. The hybrid assembly produced a sixth contig of 2440 bp. The maximum contig length represents the ~5.3 M bp chromosome, with a maximum difference of 71 bp among ONT data assemblies and 227 bp between ONT data and hybrid assembly. The total assembly length was similar between the two techniques, but in comparison, ISP data failed to assemble approximately 90,000 bases (Table 2).

### 3.2. Assembly, β-Lactamase Identification and Typing

The assemblies were queried on the same annotation pipeline (Figure 1), which included MLST typing, identification of plasmid Inc types, and resistance genes. The *K. pneumoniae* VS17 was typed as an ST16 strain, which was unambiguously identified by all methods. In all assembly methods, we also identified the presence of the following plasmid Inc types: Col (pHAD28), Col440II, IncFIB (pNDM-Mar), IncHI1B (pNDM-MAR), IncFII, and IncX3 (Table 3). Given the Inc type locations and contig sizes on the ONT-derived analyses, we determined the presence of a 5.3 Mb chromosome and ~350 kb IncFIB-IncHI1B, ~76 kb IncFII, ~45 kb IncX3, and ~5 kb Col440II circular plasmids. The large range of plasmid sizes obtained (~5 kb–~350 kb) suggests we captured all plasmids present in the samples. The lack of discrepancies among techniques suggests the absence of plasmid loss. These plasmid types are commonly found across Enterobacterales species and might carry ARGs or not.

**Figure 2:**
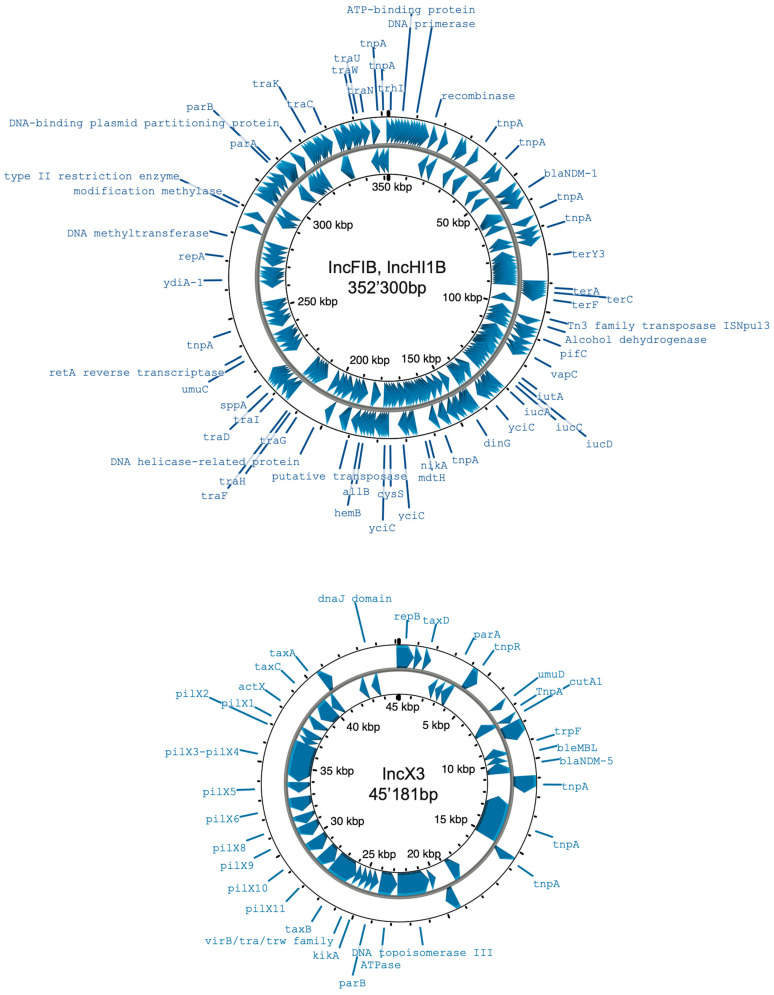
Annotated plasmid maps of type IncFIB-IncHI1B bearing *bla*_NDM-1_ (**top**) and IncX3 bearing *bla*_NDM-5_ (**bottom**).

For β-lactamase resistance gene identification and typing, *K. pneumoniae* VS17 carried a constitutive chromosomal *bla*_SHV_ gene that was identified in all assemblies, as expected. This was a new allele with 99.88% identity with *bla*_SHV-26_, its closest allele (Table 4). We could determine the chromosomal location of the *bla*_SHV_ gene from all data sets (in the short-read-only assembly, it was identified in a >600 kb contig, which would indicate it is on the chromosome). In addition, we identified *bla*_NDM-4_ with 100% identity in a ~3 kb contig of the ISP data, whose location (plasmid versus chromosomal) could not be deduced. In contrast, the hybrid assembly, as well as the long-read-only assemblies, identified two different *bla*_NDM-1_ and *bla*_NDM-5_ alleles in two separate plasmids typed as IncFIB-IncHI1B and IncX3, respectively. The IncFIB-IncHI1B and IncX3 plasmids are commonly associated with *bla*_NDM-1_ and *bla*_NDM-5_, respectively [14,15]. These NDM-producing plasmids were extracted and annotated as shown in Figure 2. Finally, the *bla*_TEM-1B_ gene was identified in contig 39 (~8 kb) but could not be typed from the ISP data; all other assemblies identified the gene in an IncFII plasmid of ~76 kb (Table 4).

### 3.3. bla_NDM_ Alleles Confirmation

**Figure 3:**
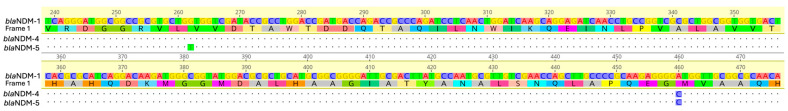
Alignment of *bla*_NDM-1_, *bla*_NDM-4_, and *bla*_NDM-5_ region where the SNPs are presents. Dots represent a perfect match at a given position.

In the context of the discrepancy between *bla*_NDM_ alleles identified by each sequencing technique, we performed (i) PCR specifically amplifying the two *bla*_NDM_ alleles (outflanking primers based on long-read sequencing sequences) followed by Sanger sequencing that confirmed the presence of the two alleles, *bla*_NDM-1_ and *bla*_NDM-5_, but failed to confirm the *bla*_NDM-4_ presence, and (ii) conjugation into *E. coli* J53. The meropenem-resistant *E. coli* transconjugant was confirmed by sequencing to carry the IncFIB-IncHI1B 352,326 bp circular plasmid harboring *bla*_NDM-1_ only. We did not obtain transconjugants for the IncX3 plasmid. Altogether, these experiments confirmed the allele identification of long-read/hybrid assemblies, and the absence of the bona fide *bla*_NDM-4_ allele. The *bla*_NDM_ alleles were aligned to depict the two SNP positions, 262GTG→TTG, V88L, and 460ATG→CTG, M154L, that differentiate these three alleles (Figure 3).

## 4. Discussion

In this work, we compared short-read, long-read, and hybrid assemblies of a complex *K. pneumoniae* genome. As expected, all methods correctly identified the MLST type; the long-read and hybrid methods accurately identified the architectural context and could circularize the chromosome and all plasmids, but short reads provided a different resistance gene identification profile than long-read-based assemblies (*bla*_NDM-4_ versus *bla*_NDM-1_ and *bla*_NDM-5_). While one could have easily presumed that this was an accuracy error of the long-read platform, we further investigated and could confirm, by specific PCR verification followed by Sanger sequencing (considered the gold standard), that long-read and hybrid assemblies were accurate. The conjugation assay also confirmed this finding. We show here (Table 3) that the short-read-only assembly produced a consensus artifact originating from the raw reads of alleles *bla*_NDM-5_ and *bla*_NDM-1_, which produced a false *bla*_NDM-4_. In this case, the two SNP positions differentiating these three alleles are 199 bp apart (Figure 3), which can cause misassembly when using short-read-only data; this issue also applies to scenarios where multiple alleles are present.

Long-read assemblies are currently being embraced as the WGS strategy that allows for rapid and accurate typing, although the ONT accuracy has been heatedly debated [16,17,18,19]. Now with an output score of Q20+, the latest chemistry seems to offer nearly as accurate results as short-read sequencing, and several groups are assessing if long-read-only strategies are suitable for cgMLST and SNP analysis. In the case of discrepancies between short-read and long-read assemblies, mainstream knowledge would favor the most accurate method. This proof-of-concept study shows that importantly, short-read assemblies’ accuracy errors, in the context of a wrong consensus artifact due to duplicate genes or presence of multiple alleles, might occur and can affect the correct identification of carbapenemase alleles.

This issue becomes evident with the high number of multiple resistance gene alleles within Enterobacterales isolates, and particularly with the increasing incidence of AMR gene duplications and double or triple carbapenemases in clinical strains identified worldwide [20,21,22,23]. There is also an obvious need for correct β-lactamase allele identification for epidemiological or infection control and contact tracing. WGS is increasingly being used in epidemiological studies and this work shows how short-read sequencing might fail to correctly identify ARG alleles. Correct typing of β-lactamase alleles is also likely to have increasingly important clinical relevance. It is now well established that specific alleles of carbapenemase genes might confer phenotypic resistance to novel β-lactam/β-lactamase inhibitor combinations, such as *bla*_KPC-113_ to ceftazidime/avibactam or *bla*_NDM-9_ to cefepime/taniborbactam [24].

Here, we identified a carbapenemase-producing *K. pneumoniae* ST16 isolate in Switzerland. This lineage, considered an emerging high-risk clone, has been increasingly reported worldwide and has been associated with severe clinical outcomes [25,26,27]. Overall, while WGS is increasingly used for Gram-negative epidemiological surveillance [28], comparing the pros and cons of each sequencing technique becomes paramount. Long-read-based surveillance allowing genomic architecture assessment and plasmid characterization is increasingly necessary [29]. The accuracy of ONT long-read sequencing reaches quality score levels that allow allele typing and possibly core genome MLST analysis, but further studies are still needed.

## Figures and Tables

**Table 1:** Raw read statistics and QC values for ISP short- and ONT long-read technologies. Read quality is given as Phred score.

Data Set	num_read	mean_len	min_len	med_len	max_len	N50	total_len	ambiguous_bp	Coverage	avg_read_quality
Illumina R1	6,451,108	100	100	100	100	100	645,110,800	5704	234.59	39.31
Illumina R2	6,451,108	100	100	100	100	100	645,110,800	70,826	*	38.15
ONT	630,578	3047.7	100	1299	232,397	6802	1,921,804,262	0	349.42	29.61

* Illumina coverage considers both strands (R1 and R2).

**Table 2:** Resulting statistics from the four assemblies obtained for the *K. pneumoniae* VS17 isolate.

Assembly	num_contig	total_assembly_length	max_contig_len	min_contig_len	num_ambig_bp	N50
Illumina_spades_contigs	277	5,762,046	325,640	56	0	152,157
Illumina_spades_scaffolds	250	5,764,032	658,511	56	2610	235,693
ONT_flye	5	5,850,368	5,371,556	5250	0	5,371,556
ONT_flye_medaka	5	5,850,437	5,371,614	5253	0	5,371,614
ONT_flye_pilon	5	5,850,448	5,371,627	5251	0	5,371,627
Unicycler (hybrid)	6	5,853,076	5,371,783	2440	0	5,371,783

**Table 3:** Summary of plasmid types obtained from the four assemblies. The contig location and identity percentage are shown in parenthesis.

Assembly	Col (pHAD28)	Col440II	IncFIB (pNDM-Mar)	IncHI1B (pNDM-MAR)	IncFII	IncX3
Illumina_spades	contig 61 (92.25)	contig 47 (100)	contig 27 (99.54)	contig 22 (99.47)	contig 26 (100)	contig 29 (100)
Unicycler	contig 6 (92.25)	contig 5 (100)	contig 2 (99.54)	contig 2 (99.47)	contig 3 (100)	contig 4 (100)
ONT_flye	contig 6 (92.37)	contig 6 (100)	contig 3 (99.54)	contig 3 (99.47)	contig 4 (100)	contig 1 (100)
ONT_flye_medaka	contig 6 (92.37)	contig 6 (100)	contig 3 (99.54)	contig 3 (99.47)	contig 4 (100)	contig 1 (100)
ONT_flye_pilon	contig 6 (92.37)	contig 6 (100)	contig 3 (99.54)	contig 3 (99.47)	contig 4 (100)	contig 1 (100)

**Table 4:** Summary of the β-lactamase resistance gene identification for the four assemblies. Different NDM alleles are shown in bold.

Assembly	MLST	Resistance Gene ^†^	Identity (%)	Contig	Size (bp)	Coverage	Circular	Location ^§^
Illumina_spades	ST16	**blaNDM-4**	100	contig 57	3036	295	no	n.d.
		blaSHV-26 *	99.88	contig 1	658,511	79	no	chromosome
		blaTEM-1B	100	contig 39	8779	218	no	n.d.
Unicycler_hybrid	ST16	**blaNDM-1**	100	contig 2	352,331	1.37× ^	yes	IncFIB, IncHI1B
		**blaNDM-5**	100	contig 4	45,181	1.42× ^	yes	IncX3
		blaSHV-26 *	99.88	contig 1	5,371,783	1.00× ^	yes	chromosome
		blaTEM-1B	100	contig 3	76,090	1.56× ^	yes	IncFII
ONT_flye	ST16	**blaNDM-1**	100	contig 3	352,292	468	yes	IncFIB, IncHI1B
		**blaNDM-5**	100	contig 1	45,181	844	yes	IncX3
		blaSHV-26 *	99.88	contig 2	5,371,556	283	yes	chromosome
		blaTEM-1B	100	contig 4	76,089	864	yes	IncFII
ONT_flye_medaka	ST16	**blaNDM-1**	100	contig 3	352,300	468	yes	IncFIB, IncHI1B
		**blaNDM-5**	100	contig 1	45,181	844	yes	IncX3
		blaSHV-26 *	99.88	contig 2	5,371,614	283	yes	chromosome
		blaTEM-1B	100	contig 4	76,089	864	yes	IncFII
ONT_flye_pilon	ST16	**blaNDM-1**	100	contig 3	352,300	468	yes	IncFIB, IncHI1B
		**blaNDM-5**	100	contig 1	45,181	844	yes	IncX3
		blaSHV-26 *	99.88	contig 2	5,371,627	283	yes	chromosome
		blaTEM-1B	100	contig 4	76,089	864	yes	IncFII

† Only beta-lactamases are described here. * New allele, closest allele is shown. n.d. cannot be determined. § Chromosome or plasmid Inc type is shown. ^ Coverage depth normalized with the chromosome sequencing depth (1.0).

## Data Availability

WGS data are available under BioSample SAMN43042374.
